# TLR5 Supports Development of Placental Labyrinthine Zone in Mice

**DOI:** 10.3389/fcell.2021.711253

**Published:** 2021-07-28

**Authors:** Jensen H. C. Yiu, Samson W. M. Cheung, Jieling Cai, Kam-Suen Chan, Jing Chen, Lai Yee Cheong, Hau-Tak Chau, Aimin Xu, Raymond H. W. Li, Connie W. Woo

**Affiliations:** ^1^State Key Laboratory of Pharmaceutical Biotechnology, Li Ka Shing Faculty of Medicine, The University of Hong Kong, Hong Kong, China; ^2^Department of Pharmacology and Pharmacy, Li Ka Shing Faculty of Medicine, The University of Hong Kong, Hong Kong, China; ^3^Department of Medicine, Li Ka Shing Faculty of Medicine, The University of Hong Kong, Hong Kong, China; ^4^Department of Obstetrics and Gynecology, Li Ka Shing Faculty of Medicine, The University of Hong Kong, Hong Kong, China

**Keywords:** CREB, labyrinthine zone, mTOR, placenta, TLR5

## Abstract

Toll plays an important role in innate immunity and embryonic development in lower-ranked animals, but in mammals, the homolog toll-like receptors (TLR) are reported to facilitate postnatal development of immunity only. Here, we discovered a role of TLR5 in placental development. *Tlr5* was highly transcribed during the placenta-forming and functional phases. TLR5 deletion led to a smaller placental labyrinthine zone and lower embryo weight, and the smaller size of embryo was overcorrected, resulting in a higher postnatal body weight. Examination of TLR5-deficient conceptus revealed a decrease in nuclear cAMP-response element-binding protein (CREB), mechanistic target of rapamycin (mTOR) and insulin growth factor-1 receptor (IGF1R) abundances in the placenta-forming phase. Non-flagellin-based TLR5 ligands were detected in serum of female mice and the overexpression of TLR5 alone was sufficient to induce CREB nuclear translocation and mTOR transcriptional activation in trophoblasts. Taken together, we uncovered the participation of TLR5 in the early placental formation in mice, unveiling a role of TLR in embryonic development in higher-ranked animals.

## Introduction

Toll or toll-like receptors (TLR) are ancient proteins present in wide ranks of organisms from Cnidaria to Chordata. In addition to the well-known functions in immunity, toll and TLR are found to mediate development in the lower-ranked animals such as *Caenorhabditis elegans* and *Drosophila melanogaster* (Leulier and Lemaitre, 2008). Deletion of toll in these organisms causes dorsalized embryos and larvae, and in *C. elegans* even leads to lethality. In mammals, ablation of TLR impedes immune defense upon microbial attacks, but no embryonic morphological abnormality or lethality has been reported. Despite that TLR is shown to regulate differentiation of adult neural and mesenchymal stem cells, the evidence of the participation of TLR in general development of higher-ranked animals such as mammals is lacking (Grasselli et al., 2018). Nonetheless, TLR family members are detected at the fetomaternal interface in eutherians, and thought to play a part in the immunological defense against potential infections (Koga and Mor, 2010). As for human placenta, TLR is expressed in the inner villous cytotrophoblast layer as early as the 6th–7th week of gestation (Pudney et al., 2016). Interestingly, robust spatiotemporal variations in the expression patterns have been reported for different TLR family members (Beijar et al., 2006; Koga and Mor, 2010) and such variations appear to be redundant if TLR only exerts immunological functions. Whether TLR family also plays a role in embryonic development in higher-ranked animals, hence, remains to be clarified.

Placentation happens solely in mammals, implicating the reliance on maternal resources during fetal development, as placenta is where the gas, nutrient and waste exchanges between the mother and fetus take place. Human and murine placentas are structurally different but share substantial similarities in terms of the molecular events during the initiation of placenta formation and functions, in particular, at the fetomaternal interface (Hemberger et al., 2019; Gerri et al., 2020). Murine placenta can be divided into decidua, junctional zone and labyrinthine zone. Labyrinthine zone is functionally analogous to chorionic villi in human, where the maternal blood flows in and directly contacts with syncytiotrophoblast cells thereby facilitating the fetomaternal exchange (Hemberger et al., 2019). In mice, the allantois extends from the mesoderm of embryo and fuses with the basal membrane of the chorionic layer from the trophectoderm, which together found the labyrinthine zone, at day 8.5 of gestation, and the structure of the labyrinthine zone is consolidated by day 11.5. In human, chorionic villus development takes place during the 4th–6th week of gestation. Syncytiotrophoblasts and cytotrophoblasts initially form finger-like extensions inserting into maternal decidua, followed by the growth of extraembryonic mesoderm into villous structures that cover the entire surface of chorionic sac (Cross et al., 2003; De Clercq et al., 2019). To facilitate the placental nutrient exchange, the mechanistic target of rapamycin (mTOR) signaling pathway is essential for both human and mice (Roos et al., 2007, 2009a, b). mTOR pathway serves as the central regulator of mammalian growth and metabolism, and its impairment in fetal compartment results in underdevelopment of labyrinthine zone and intrauterine growth restriction (IUGR) (Roos et al., 2009b; Lopez-Tello et al., 2019). Although small for gestational age newborns tend to catch up on the body weight during infancy, epidemiological studies demonstrate that overcorrection increases the risk of obesity and related morbidities in adulthood for reasons thought to be caused by intrauterine preprogramming (Hong and Chung, 2018).

In this study, we found that deletion of TLR5 in mice impaired the development of labyrinthine zone and downregulated the mTOR/S6K/IGF1R pathway in placenta. The proper development of placenta relied on the expression of TLR5 from the fetal side of the fetomaternal interface. TLR5 pathway was presumed to be constitutively active at placenta because of the constant presence of endogenous TLR5 ligands in maternal circulation. Our findings suggest that TLR pathway, in addition to immunity, also participates in the general development of placental animals.

## Materials and Methods

### Animal Models

C57BL/6J wild type, *Tlr4*^–/–^ (also known as C3H/HeJ, Stock No. 000659), *Tlr5*^–/–^ (Stock No. 008377) and C3H/HeOuJ (control for C3H/HeJ mice, Stock No. 000635) mice were purchased from the Jackson Laboratory (ME, United States) and maintained in specific-pathogen-free (SPF) environment on a 12:12 h day:night cycle with free food and water access at Laboratory Animal Unit in the University of Hong Kong. Ethics approval for all animal experimental procedures were obtained from the Committee on the Use of Live Animals for Teaching and Research of the University of Hong Kong. *Tlr5*^–/–^ mice were backcrossed with C57BL/6J wild type mice for 10 generations, and all the experiments then used the backcrossed *Tlr5*^–/–^ mice obtained from homozygous breeding. All mice were randomized prior to experiments by simple randomization. Virgin female mice were used for maternal experiments and all offspring of both genders were included in the analysis. The weights of mice were monitored over the course of pregnancy and the blood glucose was measured with Accu-Check glucometer (Roche, Switzerland). Upon sacrifice, mice were euthanized and blood was collected via cardiac puncture. Tissues were weighted, snap-frozen in dry ice and stored at –80°C for long-term storage.

### Histochemical and Immunohistochemical Staining

Placentas were dissected along the midline and one half were fixed in 4% paraformaldehyde and embedded in paraffin. Serial 5-μm-thick cross-sections were stained with hematoxylin and eosin for morphometric analyses. Sections were also examined for Ki67 abundance using horseradish peroxidase-based DAB staining with anti-Ki67 antibody (Cat. No. ab15580, Abcam, MA, United States) followed by counterstaining using hematoxylin.

### Gut Microbiota Analysis

Fecal DNA was extracted using the QIAamp DNA Stool Mini Kit (Qiagen, Netherlands), and subjected to whole genome shotgun metagenomic sequencing by Beijing Genomics Institute (Hong Kong, China) as previously described (Yiu et al., 2020). In brief, DNA fragments were sequenced using Illumina HiSeq 4000 (150 bp; paired-end). The raw sequence reads were processed with KneadData v0.10.0 (Harvard University, MA, United States) to remove mouse DNA contamination and sequences containing low quality or adapter sequences. Approximately 10-Gb cleaned data were obtained for each sample and analyzed for bacterial strains and abundance using MetaPhlAn v3.0.9 (Harvard University). The heatmap with hierarchical clustering and the non-metric multi-dimensional scaling (NMDS) plot were generated based on Bray-Curtis dissimilarity using hclust2 and vegan package of R, respectively. Linear discriminant analysis effect size (LefSe) was also applied to identify strains with statistically significant differences in abundance (Harvard University). To perform functional analysis, HUMAnN v3.0.0 was used and the identified gene families were subsequently regrouped by Gene Ontology (GO) mapping (Franzosa et al., 2018).

### *In vivo* Gut Permeability Assay

Mice were fasted for 6 h and orally gavaged with FITC-dextran-4 (0.2 mg/g, Sigma-Aldrich, MO, United States). An hour later, blood was collected from tail vein and fluorescent signals in serum were detected with CLARIOSTAR spectrometer (BMG Labtech, Germany) with an excitation wavelength of 485 nm and an emission wavelength of 535 nm to determine the level of penetrated FITC-dextran in the circulation.

### Detection of Bacterial Products

Lipopolysaccharide levels in serum and tissues were measured with limulus amoebocyte lysate assay (HyCult Biotech, Netherlands). Flagellin level in tissues was determined by immunoblotting with anti-flagellin antibody (Cat. No. pab0595, Covalab, France). The levels of flagellin and non-flagellin-based TLR5 ligands in serum were determined with HEK-Blue^TM^ mTLR5 cells (InvivoGen, CA, United States). The level of non-flagellin-based TLR5 ligands was obtained by adding the anti-flagellin antibody (1:100 final) to neutralize the flagellin-induced activation of the reporter cells whilst the level of flagellin was obtained by subtracting the non-flagellin-based TLR5 ligand level from the total TLR5 ligand level. The neutralizing efficiency was confirmed by a 10-fold ablation of the flagellin standard-induced activity. The commercially available flagellin (Enzo Life Sciences, Cat. No. ALX-522-058-C010, NY, United States) was used to generate a standard curve to calculate the amount of flagellin in samples. To determine the presence of bacterial DNA in embryonic tissues, total DNA was isolated with *Quick*-DNA Tissue/Insect Miniprep Kit (Zymo Research, CA, United States), followed by PCR amplification of 16S rRNA gene using primers Eub338 and Eub518 and agarose gel electrophoresis (Supplementary Table 1) (Fierer et al., 2005). DNA isolated from *Escherichia coli* was used to generate a standard curve to calculate the amount of bacterial DNA in samples.

### Cell Culture

Human trophoblasts, BeWo cells (Cat. No. CCL-98, American Type Culture Collection), were routinely maintained in DMEM/F12K supplemented with 10% FBS. Adenovirus encoding TLR5 (Ad-TLR5) was constructed by Welgen Inc. (MA, United States) as previously described (Yiu et al., 2020). In brief, murine *Tlr5* cDNA (GE-Dharmacon, Lafayette, United States) was inserted into pEntCMV-Ef1aGFP vector followed by adenovirus packaging in HEK293 cells. To overexpress TLR5, BeWo cells were infected with Ad-TLR5 or control adenovirus encoding green fluorescent protein (Ad-GFP, Welgen) overnight at 5 × 10^8^ viral particles/mL.

### Western Immunoblotting and Enzyme-Linked Immunosorbent Assays

Immunoblotting was conducted as described previously (Yiu et al., 2020). Primary antibodies, anti-Akt (Cat. No. 9272), anti-phospho-Akt (Cat. No. 4060), anti-CREB (Cat. No. 9104), anti-c-Jun (Cat. No. 9165), anti-GAPDH (Cat. No. 2118), anti-mTOR (Cat. No. 2983), anti-p65 (Cat. No. 8242), anti-S6K (Cat. No. 9202), anti-phospho-S6K (Cat. No. 9234), anti-S6 (Cat. No. 2317), anti-phospho-S6 (Cat. No. 2211) and anti-IGF1R (Cat. No. 3027) were purchased from Cell Signaling Technologies (MA, United States). Anti-4EBP (Cat. No. 6936) and anti-phospho-4EBP (Cat. No. 12884) were purchased from Santa Cruz Biotechnology (CA, United States). Anti-lamin B1 (Cat. No. 12987-1-AP) was purchased from ProteinTech (IL, United States). Anti-β-actin (Cat. No. A1978) was purchased from Sigma-Aldrich. The membranes were probed with anti-β-actin, anti-GAPDH or anti-lamin B1 antibodies for detecting differences in protein loading. The horseraddish peroxidase-conjugated secondary antibodies were purchased from Jackson ImmunoResearch Laboratories Inc. (PA, United States). Densitometry analyses of the bands were carried out using Image J software developed by National Institutes of Health (MD, United States). CCL2 (Cat. No. DY479), IGF1 (Cat. No. DY791), IGF2 (Cat. No. DY792), IL1β (Cat. No. DY401), IL6 (Cat. No. DY406), and TNFα (Cat. No. DY410) enzyme-linked immunosorbent assay (ELISA) kits were purchased from R&D Systems (MN, United States).

### Chromatin Immunoprecipitation

Chromatin from conceptus was prepared and sheared by sonication with Bioruptor^®^ (Diagenode, Belgium) as previously described (Yiu et al., 2020), followed by immunoprecipitation with anti-CREB antibody (Cat. No. 9197, Cell Signaling Technology) or rabbit IgG isotype (Cat. No. 3900, Cell Signaling Technology) and protein-A/G magnetic beads (Bimake, TX, United States). DNA precipitated from the samples was subjected to PCR amplification detecting a segment of *Mtor* promoter region using the primers, 5′-TGGTGGCACAGTCTAAAGAAA-3′ (forward) and 5′-CAATTCCCAGCACTGTGTAAAG-3′ (reverse).

### Real-Time PCR and Droplet Digital PCR

Total RNA from tissues or cells was isolated RNeasy Mini Kit (Qiagen). RNA was reverse-transcribed into cDNA using ImProm-II^TM^ Reverse Transcription System (Promega, WI, United States). Real-time PCR was conducted using the SYBR green PCR reagent (Roche) and StepOnePlus^TM^ System (Applied Biosystems, CA, United States). As for droplet digital PCR, it was performed on QX200 Droplet Digital PCR system (Bio-Rad, CA, United States) according to the manufacturer’s instruction. In brief, 20 μl reaction mixture containing cDNA template equivalent to 5–30 ng RNA, 150 nM primers and ddPCR EvaGreen Supermix (Bio-Rad) was partitioned into more than 10,000 droplets with QX200 Droplet Generator (Bio-Rad) and amplified using C1000 Touch Thermal Cycler (Bio-Rad). The fluorescent signals were then analyzed with QX200 Droplet Reader and QuantaSoft Software (Bio-Rad). Samples with QuantaSoft Quality Scores below 0.85 or containing fewer than 8,000 droplets were excluded. The sequences of the primers are listed in Supplementary Table 1.

### Statistical Analysis

Statistical analyses were performed using IBM SPSS Statistics Version 23.00 (IBM Corporation, NY, United States) or R software Version 4.0.5. A power of 90% was achieved for this study, on the two independent group design, 5% significance, and the difference of 20% in placetal labyrinthine thickness with standard deviation of 85 μm. Data are represented as mean ± standard error of mean (SEM). The unpaired Student’s *t*-test and Mann Whitney U test were applied for two group comparison with and without normal distribution, respectively. *P*-values were adjusted with Benjamini-Hochberg method for multiple comparison in Figure 2C. The difference in gut microbiota pattern was evaluated by the Permutational Analysis of variance (PERMANOVA) using the adonis function from the vegan package on R. *P*-values less than 0.05 were accepted to indicate statistically significant differences.

## Results

### TLR5 Deficiency Leads to the Thinning of Placental Labyrinthine Zone

**FIGURE 1:**
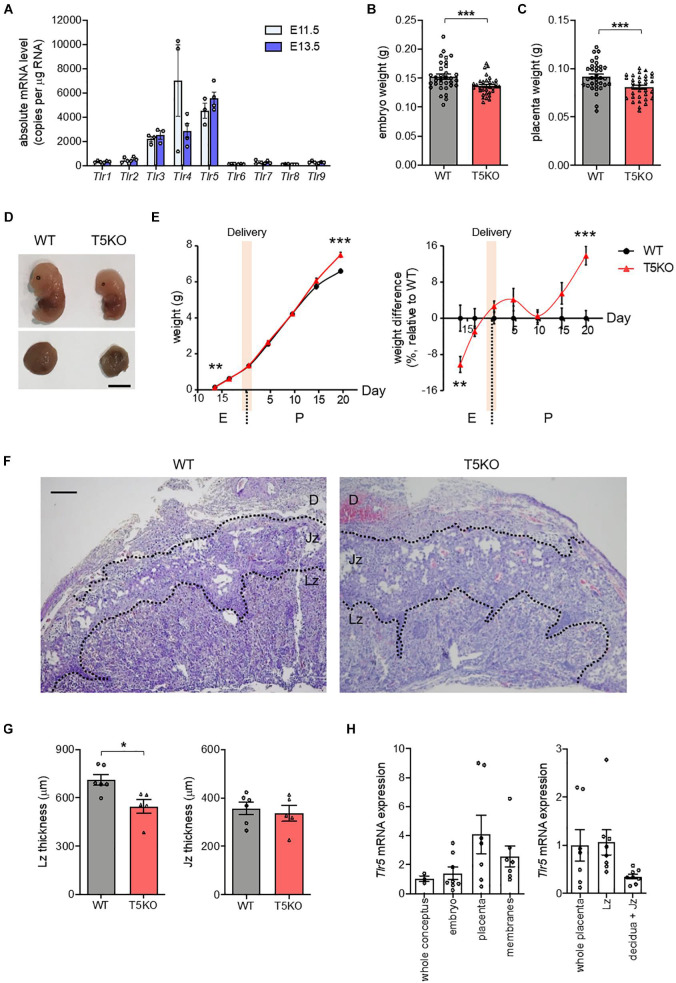
TLR5 deficiency compromised placental labyrinthine development. **(A)** Mated wild type (WT) C57BL/6J female mice were sacrificed at indicated gestational days. Presence of vaginal plug was defined as embryonic day 0.5 (E0.5). Absolute copy numbers of mRNA transcripts in E11.5 and E13.5 placenta were determined using droplet digital PCR (*n* = 3–4 litters, one randomly picked placenta from each litter). **(B–D)** Pregnant WT and TLR5-knockout (T5KO) mice were sacrificed at E13.5 (*n* = 33–34 embryos from 5 litters). **(B)** Embryo and **(C)** placenta weights were measured. **(D)** Photographs of representative embryos and placentas are shown. Scale bar is 5 mm. **(E)** Embryo and pup weights from E13.5 to postpartum day 20.5 (P20.5, weaning) were recorded. The body weights relative to wild type controls were shown on the right panel. The shaded area indicates the delivery periods (*n* = 33–34 embryos from 5 litters for E13.5; *n* = 33–35 embryos from 4 litters for E16.5; *n* = 38–39 pups from 4–5 litters for P0.5; *n* = 25–33 pups from 4 litters for P4.5; *n* = 31–37 pups from 4 litters for P9.5; *n* = 25–31 pups from 4 litters for P14.5; *n* = 40–41 pups from 5 litters for P19.5). **(F,G)** Sections of E13.5 placentas were subjected to hematoxylin-eosin staining. **(F)** Representative images are shown. Scale bar is 100 μm. Maternal decidua (D), junctional zone (Jz) and labyrinthine zone (Lz) are denoted. **(G)** The thicknesses of Lz and Jz were measured. Each dot represents the average thickness for one litter (*n* = 5–6 litters). **(H)** The relative *Tlr5* mRNA expression at different compartments in WT (left panel) conceptus and (right panel) placenta at E13.5 was examined. *Rn18s* was used as the housekeeping control (*n* = 8 conceptuses). Data are represented as mean ± SEM. The differences between WT and T5KO mice were determined by two-tailed Student’s *t*-test. *P < 0.05, **P < 0.01, ***P < 0.001.

TLR1 to TLR9 are commonly expressed in human and mice. The absolute mRNA copy numbers for these TLR in the placenta from pregnant wild type C57BL/6J mice at embryonic day 11.5 (E11.5) and E13.5, the period during which the placenta became mature and fully functional (Cross et al., 2003), were determined using droplet digital polymerase chain reaction. The abundance of *Tlr5* mRNA was the highest in both E11.5 and E13.5 placentas, followed by *Tlr3* and *Tlr4* mRNA (Figure 1A). Therefore, we examined the pregnancy in *Tlr5*^–/–^ mice in order to identify the role of placental TLR5. Wild type and *Tlr5*^–/–^ mothers had similar weight gains throughout pregnancy (Supplementary Figure 1A), despite that heavier weight in *Tlr5*^–/–^ mice was previously reported (Vijay-Kumar et al., 2010). Fat percentage and fasting blood glucose level were also comparable between pregnant wild type and *Tlr5*^–/–^ mice (Supplementary Figures 1B,C). We then examined the embryonic weights, and no difference in the weights of conceptus was observed between both genotypes at E7.5 to E9.5 (Supplementary Figure 1D). At E11.5, the formation of a definite placenta was underway, and the weights of embryo and placenta were separately measured starting from then. No difference in the weights of both embryo and placenta between the two genotypes was observed at E11.5 (Supplementary Figure 1E). However, at E13.5, TLR5 deletion led to lower embryonic and placental weights (Figures 1B–D). Then, at E16.5 when the liver of embryo matured and placenta began to degenerate (Xue et al., 2013), both embryo and placenta weights in *Tlr5*^–/–^ mice caught up with those in wild type (Supplementary Figure 1E). We further tracked the postnatal body weight change and found that the *Tlr5*^–/–^ pups became heavier as the weaning age approached (Figure 1E). Overall, deletion of TLR5 did not affect the litter size and embryo survival (Supplementary Figures 1F,G). Next, we examined whether there was any morphological deformity in placenta potentially contributing to the decreased weights of placenta and embryo at E13.5. A significantly thinner labyrinthine zone was observed in *Tlr5*^–/–^ placenta compared with wild type mice (Figures 1F,G). Moreover, placenta had the highest TLR5 expression within the conceptus and a higher expression of TLR5 was detected in the labyrinthine zone rather than junctional zone (Figure 1H), prompting us to anticipate a supportive role of TLR5 in the labyrinthine zone.

As the amount of *Tlr4* mRNA transcript was comparable to that of *Tlr5* at E11.5 (Figure 1A), we also investigated whether TLR4 was similarly involved in the development of placenta using C3H/HeJ mice, which carried a missense point mutation in the *Tlr4* gene that resulted in hyporesponsiveness to ligands such as lipopolysaccharide (LPS). Unlike *Tlr5*^–/–^ mice, there was no difference in the placenta weight or the thickness of labyrinthine zone between the *Tlr4*^–/–^ mice and their wild type littermates, and *Tlr4*^–/–^ embryos were slightly heavier (Supplementary Figure 2). Considering the high TLR5 expression in mid-pregnancy and the placental defect in *Tlr5*^–/–^ mice, it is likely that the participation of TLR in the placental and embryonic development is unique to TLR5.

### Pregnancy Does Not Alter the Gut Bacteria-Derived TLR Ligands and Flagellin

**FIGURE 2:**
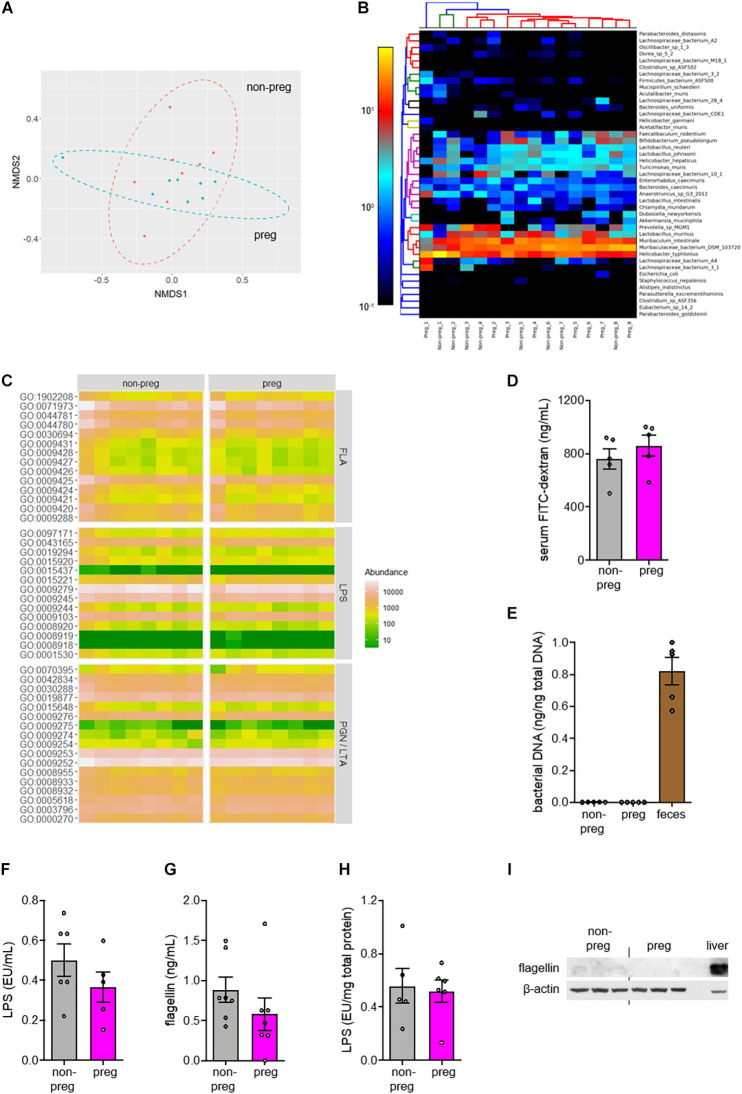
No change in β-diversity or functional pathways related to TLR ligands in gut microbiota was observed during pregnancy. **(A–C)** Feces were collected from wild type non-pregnant (non-preg) and pregnant (preg) mice at embryonic day 9.5 and the isolated DNA was subjected to shotgun metagenomic sequencing (*n* = 8 mice). **(A)** The non-metric multi-dimensional scaling (NMDS) plot of species-level relative abundances using the Bray-Curtis dissimilarity index was shown. **(B)** The heatmap shows the relative abundance of identifiable bacterial strains present in feces from both groups. The number following the underscore indicates the sample number. The differences in composition between groups were determined by PERMANOVA (*R*^2^ = 0.0287, *P* = 0.804, *df* = 15). **(C)** The relative abundances of genes involved in biosynthesis of flagellins (FLA), lipopolysaccharides (LPS), peptidoglycans (PGN) and lipoteichoic acids (LTA) were shown. **(D)** Wild type non-pregnant and pregnant mice were fasted for 6 h and orally gavaged with FITC-conjugated dextran. Gut permeability was assessed by the measurement of fluorescent signals of FITC in serum (*n* = 5 mice). **(E)** Total DNA was isolated from uterus of non-pregnant mice and conceptus from pregnant mice (*n* = 5–6 mice). The amount of bacterial DNA was quantified by 16S rRNA gene amplification. Feces was included as positive control. **(F–I)** The serum levels of **(F)** LPS and **(G)** flagellin were measured. The conceptual levels of **(H)** LPS and **(I)** flagellin were evaluated with uterus as baseline for the non-pregnant group (*n* = 5–7 mice). Liver was included as positive control for flagellin immunoblotting. Representative images are shown. Data are represented as mean ± SEM. The differences between non-pregnant and pregnant groups were determined by two-tailed Student’s *t*-test. *P*-values were adjusted for multiple comparison in **(C)**.

Recent studies have suggested various roles of gut microbiome in pregnancy (Koren et al., 2012; Gomez de Aguero et al., 2016), whereas TLR5, a receptor recognizing flagellin, helps control the growth of flagellated bacteria in the gut (Cullender et al., 2013). We speculated that the supportive role of TLR5 in placental development might be associated with gut microbiota. From the shotgun metagenomic analysis on feces collected from non-pregnant mice and pregnant mice at E9.5 when labyrinthine zone was forming (Cross et al., 2003), we did not observe any distinctive difference in the non-metric multidimensional scaling plot of species-level relative abundances using the Bray-Curtis dissimilarity index (Figure 2A and Supplementary Dataset 1). Although an elevation in the abundance of various strains of *Lactobacillus* during pregnancy was observed (Figure 2B), it did not pass the linear discriminant analysis effect size analysis. Furthermore, no difference was observed when the identifiable strains were classified according to Gram staining or flagellation (Supplementary Figure 3). These results agreed with the previous study reporting that the gut microbiota from pregnant mice at mid-gestation have a phylogenetic profile similar to that in non-pregnant mice (Gohir et al., 2015). In addition, the abundances of genes representing the biosynthesis of TLR ligands, namely peptidoglycan, lipoteichoic acid (TLR2 ligands), LPS (TLR4 ligand) and flagellin (TLR5 ligand), were also unaltered by pregnancy (Figure 2C and Supplementary Dataset 2). However, the absence of change in the abundance of genes involved in the synthesis of TLR ligands in bacteria could not rule out the possible altered penetration of these bacterial products. To this end, we evaluated the gut permeability at the mid-phase of pregnancy, but no alteration was observed (Figure 2D). Moreover, neither the uterus nor the conceptual tissues were detected with bacterial ribosomal DNA gene (Figure 2E and Supplementary Figure 4), which was congruent to the finding of Kuperman et al. (Kuperman et al., 2020). In line with the intact gut, the circulating LPS and flagellin levels remained unchanged upon pregnancy (Figures 2F,G). We detected a low level of LPS but a negligible level of flagellin in the conceptus when comparing with uterus from the non-pregnant group (Figures 2H,I). The detected LPS level was likely a reflection of that in blood. In summary, our data show that the action of TLR5 during pregnancy is not associated with any alteration in the level of gut bacteria-derived TLR ligands.

### Development of Labyrinthine Zone Depends on the TLR5 From Embryos

**FIGURE 3:**
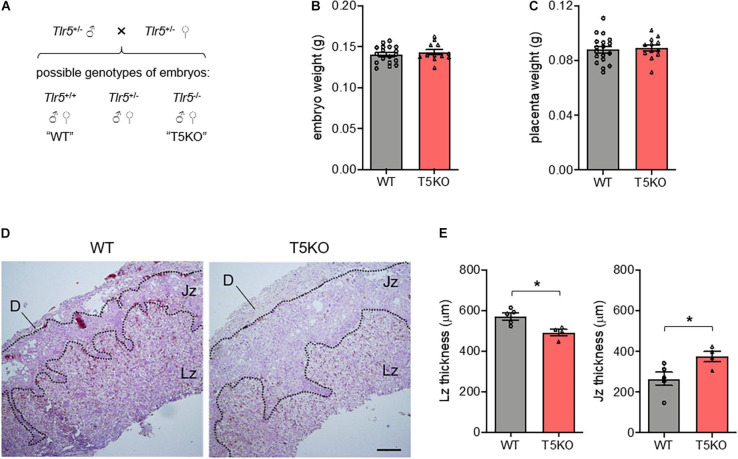
The proper development of labyrinthine zone relied on the fetal expression of TLR5. **(A)** A schematic diagram depicting possible outcomes of fetal genotypes, namely *Tlr5*^+/+^ (wild type, WT), *Tlr5*^–/–^ (TLR5-knockout, T5KO) and *Tlr5*^+/–^ (heterozygous T5KO) is shown. Heterozygous *Tlr5*^+/–^ mice were mated and sacrificed at embryonic day 13.5. Genotypes of individual embryos were determined. **(B,C)** The **(B)** embryo and **(C)** placenta weights were measured (*n* = 18 embryos for WT, *n* = 12 embryos for T5KO, from 5 litters). **(D,E)** Placenta sections were subjected to hematoxylin-eosin staining. **(D)** Representative images are shown. Scale bar is 200 μm. Maternal decidua (D), junctional zone (Jz) and labyrinthine zone (Lz) are denoted. **(E)** Thicknesses of Lz and Jz were measured. Each dot represents the average thickness for one litter (*n* = 5 litters). Data are represented as mean ± SEM. The differences between WT and T5KO mice were determined by two-tailed Student’s *t*-test. *P < 0.05.

As the mouse placenta comprises maternal cells such as the decidua and fetal cells including trophoblasts, we tried to pin down the TLR5 of which contributed to the proper labyrinthine zone development. Mating heterozygous *Tlr5*^+/–^ parents was able to yield wild type and homozygous TLR5-deficient offspring in the same litter (Figure 3A). Under this mating strategy, although there was no difference in embryo and placenta weights between wild type and *Tlr5*^–/–^ embryos generated from the same parents (Figures 3B,C), the *Tlr5*^–/–^ embryos reproduced the thinner placental labyrinthine zone phenotype compared with the TLR5-intact littermates (Figures 3D,E). The junctional zone of the placenta of *Tlr5*^–/–^ embryos was thicker than that of wild type littermates. On the other hand, TLR5 signaling has been reported to induce proliferation of fibroblasts (Hasan et al., 2005) and normal development of placenta involves proliferation of trophoblasts (Muhlhauser et al., 1993; Huppertz and Herrler, 2005). The thinning of labyrinthine zone in *Tlr5*^–/–^ mice might result from a decrease in proliferation of placental cells. However, no difference in the expression of Ki67, a classic marker of proliferation, was observed in the labyrinthine zone between wild type and *Tlr5*^–/–^ mice (Supplementary Figure 5). The proliferation was found to be more active in labyrinthine zone than junctional zone in general (Supplementary Figure 5B). Taken together, these data suggest that the TLR5 expressed in cells from the embryos facilitates the proper development of labyrinthine zone in the placenta and TLR5 appears to be involved in the early phase of placental formation.

### *Tlr5*^–/–^ Conceptus Shows Diminished Placental mTOR and IGF1R Expression

**FIGURE 4:**
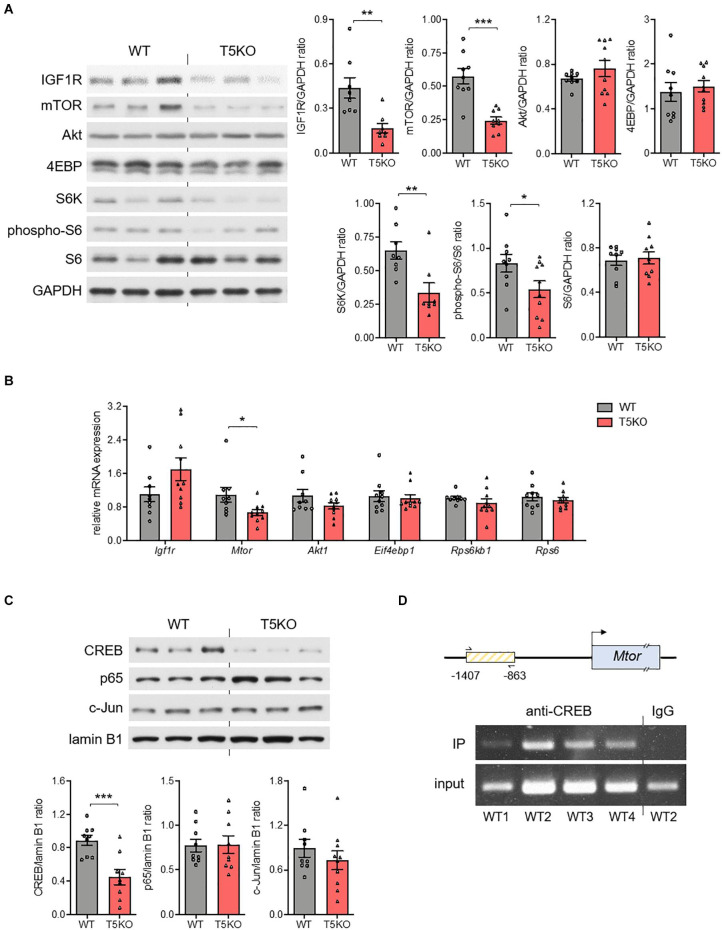
Placental mTOR pathway was regulated by TLR5. **(A–C)** Conceptuses were isolated from pregnant wild type (WT) and TLR5-knockout (T5KO) mice at day 9.5 of pregnancy (*n* = 8–10 litters, one randomly picked conceptus from each litter). The **(A)** protein and **(B)** mRNA expressions of IGF1R, mTOR, Akt, 4EBP, S6K, phospho-S6 and S6 were determined with GAPDH as loading control. **(C)** The nuclear abundances of CREB, c-Jun, and p65 were examined with lamin B1 as loading control. The densitometric analyses for A and C are shown on the right and lower panels, respectively. **(D)** Chromatin immunoprecipitation (IP) with anti-CREB antibody followed by PCR amplification using primers recognizing a segment of *Mtor* promoter [the box with yellow lines; contains the putative CREB binding site(s)] was performed with WT conceptus (*n* = 4 litters, one randomly picked conceptus from each litter). Representative images are shown. Data are represented as mean ± SEM. The differences between genotypes were determined by two-tailed Student’s *t*-test. *P < 0.05, **P < 0.01, ***P < 0.001.

The growth of placenta hinges on the insulin-like growth factor-2 (*Igf2)* transcript specifically expressed in the labyrinthine trophoblasts (Constancia et al., 2002), so we investigated whether the embryonic TLR5 regulated the placental IGF2 abundance. Since the labyrinthine zone in murine placenta started to form after the fusion of embryonic allantois and the chorion layer on E8.5 (Cross et al., 2003), the E9.5 conceptuses were used for examination of the upstream molecular events. However, no difference in the conceptual level of IGF2 between wild type and *Tlr5*^–/–^ conceptuses was observed (Supplementary Figure 6A). Similarly, there was also no difference in IGF1 level (Supplementary Figure 6B). Although the supply of the ligands was unchanged, the thinning of labyrinthine zone in *Tlr5*^–/–^ mice was possibly owing to the interruption to their receptor, IGF1 receptor (IGF1R), or its signaling pathway. Indeed, we found that the expression of IGF1R was suppressed in *Tlr5*^–/–^ mice (Figure 4A). Since mTOR signaling regulates IGF1R expression (Kwon et al., 2009), we examined the signaling activity of mTOR pathway. However, there was no difference in the phosphorylation ratio of Akt, S6 kinase (S6K) and 4E-binding protein (4EBP) between wild type and *Tlr5*^–/–^ developing conceptuses (Supplementary Figure 7). On the contrary, decreases in total protein abundances of mTOR and S6K but not 4EBP were observed in the *Tlr5*^–/–^ group (Figure 4A). Consistent with the downregulation in S6K, the phosphorylation of S6, the substrate of S6K, was also lowered by TLR5 deficiency (Figure 4A). Next, we examined the mRNA expressions of these signaling proteins and only the mRNA expression of mTOR was found suppressed in *Tlr5*^–/–^ conceptus (Figure 4B). Overall, these observations on *Tlr5*^–/–^ mice are in line with those on newborns with IUGR who often also show impairment in placental mTOR/IGF1R pathway (Roos et al., 2007; Street et al., 2011).

### Binding Element(s) of CREB Is Present in mTOR Promoter Region and TLR5 Deletion Reduces Placental IL1β Without Altering Nuclear NFκB

Several transcription factors are known to be regulated by TLR5 signaling, including nuclear factor-κB (NFκB), activator protein-1 (AP-1) and cAMP-response element-binding protein (CREB) (Rodriguez-Jorge et al., 2019). No difference in the nuclear abundances of p65 subunit of NFκB or c-Jun subunit of AP-1 but a substantial decrease in nuclear CREB abundance was observed in *Tlr5*^–/–^ conceptus (Figure 4C). The result of the chromatin immunoprecipitation suggested that a direct binding of CREB to the promoter region of *Mtor* gene took place (Figure 4D). On the other hand, earlier studies reported pleiotropic functions of several cytokines during placenta formation (Hauguel-de Mouzon and Guerre-Millo, 2006). For example, interleukin-1β (IL1β) promotes trophoblast motility whilst IL6 regulates human chorionic gonadotropin release in trophoblasts (Nishino et al., 1990; Prutsch et al., 2012). Hence, we examined whether TLR5 deletion might have altered the local cytokine profile, and found that the absence of TLR5 exclusively suppressed the placental expression of IL1β (Supplementary Figure 8). The lack of NFκB inhibition and a collective change in the cytokine profile implicates a unique placental regulation of IL1β by TLR5. Overall, the supportive role of TLR5 in development of labyrinthine zone is likely mediated by multiple mechanisms.

### Overexpression of TLR5 Alone Increases mTOR and Nuclear CREB Expressions in Human Trophoblast

**FIGURE 5:**
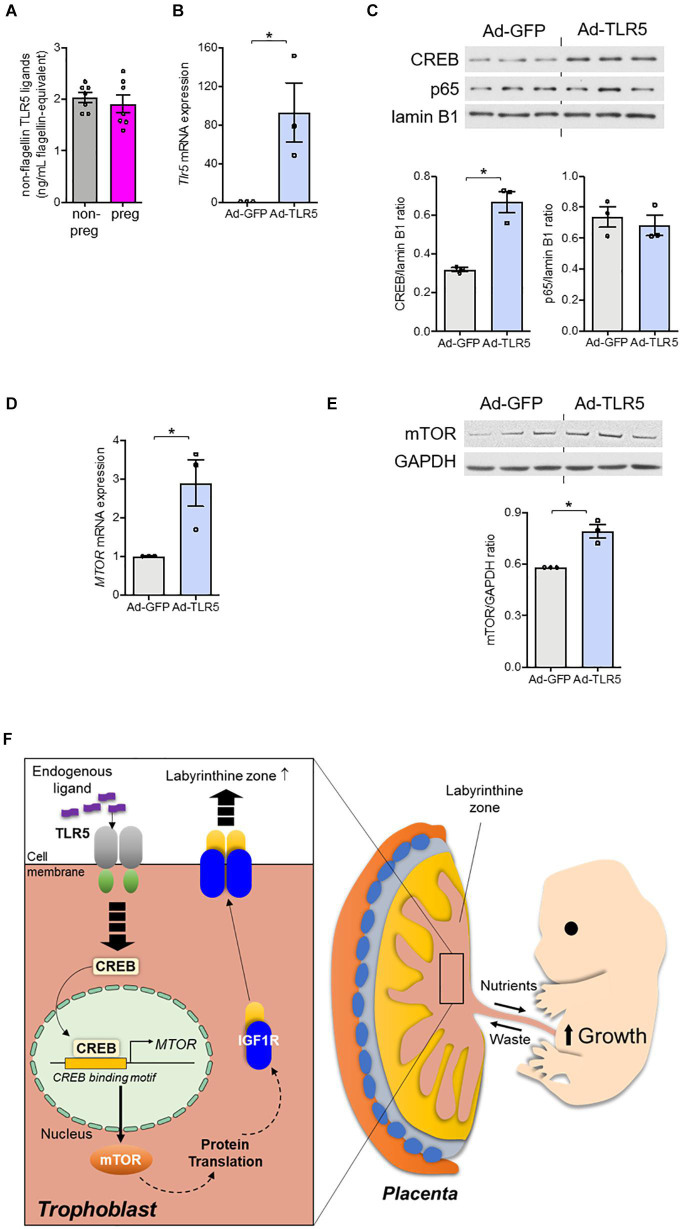
Overexpression of TLR5 alone was sufficient to upregulate mTOR expression in trophoblasts. **(A)** Serum level of non-flagellin-based TLR5 ligands in wild type non-pregnant (non-preg) and pregnant (preg) mice at day 9.5 of gestation was measured (*n* = 7 mice). **(B–E)** BeWo trophoblasts were infected with adenovirus (5 × 10^8^ viral particle/mL) encoding GFP (Ad-GFP) or TLR5 (Ad-TLR5) overnight followed by determination of **(B)** *Tlr5* mRNA expression, **(C)** nuclear CREB and p65 abundances, and mTOR **(D)** mRNA and **(E)** protein abundances (*n* = 3 independent experiments). Lamin B1 was used as loading control for nuclear protein determination and GAPDH was used for the remaining experiments. **(F)** The findings of this study are depicted. Representative results and the densitometric analyses for immunoblotting are shown. Data are represented as mean ± SEM. The differences between treatments were determined by two-tailed Student’s *t-*test. *P < 0.05.

Since the TLR5 signaling was involved, the absence of flagellin in conceptus (Figure 2I) led us to speculate the participation of endogenous TLR5 ligands (Chamberlain et al., 2012; Das et al., 2016). In line with this notion, we observed a substantial amount of non-flagellin-based TLR5 ligands in the circulation (Figure 5A). However, the circulating level of the endogenous TLR5 ligands stayed similar before and during pregnancy (Figure 5A). Therefore, we presumed that the receptor availability was a determinant of the TLR5-supported labyrinthine zone development. As a proof-of-concept experiment, we overexpressed TLR5 in human BeWo trophoblasts with adenovirus encoding TLR5 or control adenovirus encoding green fluorescent protein. The TLR5-overexpressing BeWo cells exhibited an elevated nuclear CREB abundance as well as increased expression of mTOR at both protein and mRNA levels whilst the nuclear p65 translocation was not induced (Figures 5B–E). Moreover, the detection of IL1β was negative, which was congruent to the previous suggestion that the placental cytokines are mostly secreted from immune cells (Hauguel-de Mouzon and Guerre-Millo, 2006). These data demonstrate that overexpressing TLR5 alone without altering the ligand level is sufficient to induce the nuclear translocation of CREB selectively and the subsequent transcriptional activation of mTOR. Altogether, our data provide evidence suggesting that TLR5/CREB/mTOR pathway contributes to fetal development through supporting the formation of labyrinthine zone of placenta (Figure 5F).

## Discussion

In this study, the most prominent gestational phenotype of *Tlr5*^–/–^ mice was the underdevelopment of placental labyrinthine zone. With the interdigitation of maternal and fetal blood vessels, labyrinthine zone is the key interface for fetomaternal exchange of nutrients and hormones that nourish the growing fetuses. A recent systematic screening in genetically modified mice demonstrates that defects in placental labyrinthine zone are major causes of embryonic sub-viability or even lethality (Perez-Garcia et al., 2018). In less severe cases, a thinner labyrinthine zone still leads to inadequate nutritional and hormonal supply to embryos, and, hence, smaller fetuses. Newborns with IUGR often show an impairment in placental mTOR/IGF1R pathway (Roos et al., 2007; Street et al., 2011). Along with the suppression of mTOR/IGF1R pathway and thinner labyrinthine zone, smaller embryos were also observed upon deletion of TLR5 (Figure 1), but the *Tlr5*^–/–^ embryos managed to catch up in size at E16.5 when the fetal liver matured and the fetal growth became less reliant on placenta (Supplementary Figure 1E). *Tlr5*^–/–^ mice were previously reported to exhibit hyperphagia, higher body weight and fat mass in adulthood (Vijay-Kumar et al., 2010). These suggest that the prenatal underdevelopment may affect intrauterine metabolic preprogramming. Hence, in addition to the previously reported effect of perturbed gut microbiota (Vijay-Kumar et al., 2010), the metabolic phenotypes of *Tlr5*^–/–^ mice may have also been attributed to the IUGR condition resulted from the underdeveloped placental labyrinthine zone during the gestational period. Interestingly, the junctional zone was similar in homozygous *Tlr5*^–/–^ mating but it became slightly thicker in *Tlr5*^–/–^ placenta than wild type placenta when the heterozygous mating was adopted, we speculated that the latter observation was a compensatory phenotype to avoid imbalance in blood pressure originated from the otherwise different placental sizes within the same litter.

mTOR is an evolutionarily conserved serine/threonine protein kinase known for the roles in cell proliferation, protein translation and nutrient recycling. Some studies demonstrate that TLR pathways crosstalk with mTOR pathway but the identified effects center on how mTOR modulates the inflammatory outcomes (Bodur et al., 2018; Luo et al., 2018). For instance, TLR4/PI3K-induced mTOR activation biases macrophages toward anti-inflammatory cytokine production (Luo et al., 2018), whilst endosomal TLR/TANK-binding kinase-1 (TBK1)-induced mTOR activation augments the interferon regulatory factor-3 (IRF3)-mediated interferon response (Bodur et al., 2018). In these scenarios, mTOR is regulated by TLR through the post-translational phosphorylation. Here, we show that instead of manipulating the kinase activity, TLR5 regulates mTOR protein expression at transcriptional level in trophoblasts. Nonetheless, little is known about the transcriptional regulation of mTOR, though an auto-regulatory increase in mTOR mRNA and translation has been reported in the case of nerve injury (Terenzio et al., 2018). In our model, binding site(s) for CREB was found in the promoter region of mTOR, suggesting a novel regulatory mechanism of mTOR expression. Other than mTOR, we also observed diminished protein abundances of S6K and IGF1R in *Tlr5*^–/–^ conceptus but their mRNA levels were similar to those in wild type. The suppression of S6K and IGF1R was possibly an outcome of the decreased translation secondary to mTOR downregulation. Overall, our findings put forward the notion that the crosstalk between TLR and mTOR pathways is not limited to immunological refinement.

The participation of TLR in embryogenesis is evident in several lower-ranked animals, but in mammals only the developmental roles in immunity have been reported. Immunity genes undergo positive selection frequently to accommodate host-pathogen coevolution and TLR family is suggested to be coevolved with pathogens to enhance the recognition (Wlasiuk and Nachman, 2010). However, the ligand-interacting interface remains conserved in the receptors, possibly because the molecular patterns recognized by TLR are essential for the survival of the invaders (Medzhitov and Janeway, 1997; Wlasiuk et al., 2009; Areal et al., 2011). In the case of TLR5, only 1.3% of codons show strong positive selection, and the rest including the signaling components are largely conserved (Wlasiuk et al., 2009). Nonetheless, the dominant loss-of-function polymorphism of TLR5 (TLR5^392STOP^) is present in a high percentage of population in spite of increased susceptibility to Legionnaire’s disease (Hawn et al., 2003). In fact, NOD-like receptor family CARD domain containing-4 (NLRC4) also responds to flagellated bacteria by eliciting inflammasome-mediated immunity (Zhao et al., 2011). These implicate a certain degree of functional redundancy in the immunological role of TLR5. Furthermore, it is likely that the participation of TLR5 in placental development depends on its high expression rather than a change in the ligand level. In this study, non-flagellin-based TLR5 ligands were detected in the serum of pregnant mice. Rather than coevolving along with pathogens, the conservation of TLR5 may be essential for the binding of endogenous ligands. High motility group box-1 (HMGB1), a protein released from necrotic cells, is reported to be an endogenous ligand of TLR5 (Das et al., 2016), and whether it contributes to the detected non-flagellin-based TLR5 ligands in serum requires further study. In view of the pathogen recognition by other TLR family members and NLRC4 and the heavy demand on nutrient and energy for the growth of fetus, a high transcription of conceptual TLR5 during the mid-phase of pregnancy for immunological purposes appears counterintuitive and a non-immunological role of TLR5 in pregnancy is therefore highly possible.

Similar to other prenatal studies, the failure to address tissue or cell specificity and cause-effect relationship *in vivo* is the limitation of our study. Although we provided evidence to show that the labyrinthine zone expansion is fetal TLR5- rather than maternal TLR5-dependent, it was difficult to differentiate which types of cells were involved because cells underwent active differentiation along with extensive changes in the global gene expression profile in the initial phase of placenta formation. Moreover, the existing tools for gene reconstitution themselves will unavoidably affect the embryonic development *in vivo*. Hence, we carried it out using *in vitro* setting for proof-of-concept experiments. We also attempted to identify the location of TLR5 in the conceptus and placenta, but we faced a problem in the specificity of TLR5 antibodies. Four commercially available TLR5 antibodies raised against different immunogens had been tested but none of them showed specific detection upon immunostaining (Kirkpatrick, 2009). The validation of molecular mechanisms *in vivo* requires further investigation. Nonetheless, our observation on the highly expressed TLR5 coincided with a previous study using RNA sequencing which similarly demonstrated a prominently higher expression of *Tlr5* mRNA than all other TLR members in E7.5 and E9.5 placentas (Tuteja et al., 2016). Besides TLR5, a high abundance of *Tlr3* and *Tlr4* mRNA in placenta was also detected in our experiment. Placental TLR3 and TLR4 have been previously reported to induce premature termination of pregnancy upon detection of viral and bacterial infection, respectively (Koga et al., 2009; Arce et al., 2012). Although we did not observe any obvious abnormality in *Tlr4*^–/–^ placenta, *Tlr4*^–/–^ embryos were slightly heavier than the wild type litter. Other than their latent role until the presence of infection, it is noteworthy to investigate the potential functions of TLR3 and TLR4 during development in the future.

Different cell types vary in the expression of TLR in terms of the isoform and the abundance. The expression of TLR in organs is often derived from embedded immune cells and related to immunological functions. However, TLR5 is diversely distributed in human tissues including those non-immune-related organs such as ovary, prostate, placenta and testis (Zarember and Godowski, 2002). Here, we found that deletion of TLR5 led to an impairment in the placental development that was associated with diminished mTOR and IGF1R expressions. Overexpression of TLR5 in trophoblasts, in turn, induced CREB transcription activity, resulting in stimulation of mTOR expression. Overall, the present study provides the evidence that TLR signaling is not a mere immunological pathway but its role in general development actually remains in mammals, and, hence, its biological functions should be investigated from broader perspectives.

## Data Availability Statement

The datasets presented in this study can be found in online repositories. The names of the repository/repositories and accession number(s) can be found below: SRA, accession: PRJNA732220.

## Ethics Statement

The animal study was reviewed and approved by the Committee on the Use of Live Animals for Teaching and Research of the University of Hong Kong.

## Author Contributions

JY and CW designed the experiments and prepared the manuscript. JY led and KC, JCa, SC, JCh, LC, and HC assisted the experiments. RL and AX provided critical comments on the manuscript. CW provided funding for all the experiments. All authors contributed to the article and approved the submitted version.

## Conflict of Interest

The authors declare that the research was conducted in the absence of any commercial or financial relationships that could be construed as a potential conflict of interest.

## Publisher’s Note

All claims expressed in this article are solely those of the authors and do not necessarily represent those of their affiliated organizations, or those of the publisher, the editors and the reviewers. Any product that may be evaluated in this article, or claim that may be made by its manufacturer, is not guaranteed or endorsed by the publisher.
