# Supplementary material for: TLR5 Supports Development of Placental Labyrinthine Zone in Mice
Source: Front Cell Dev Biol. 2021 Jul 28;9:711253. doi: 10.3389/fcell.2021.711253 (PMC8356041; doi:10.3389/fcell.2021.711253)
Supplement: Supplementary file 1 [file Data_Sheet_1.docx]

Supplementary Material

**TLR5 supports development of placental labyrinthine zone in mice**

Jensen H. C. Yiu^1,2,*^, Samson W. M. Cheung^1,2^, Jieling Cai^1,2^, Kam-Suen Chan^2^, Jing Chen^1,2^, Lai Yee Cheong^1,2^, Hau-Tak Chau^1,3^, Aimin Xu^1,2,3^, Raymond H. W. Li^4^, Connie W. Woo^1,2,*^

^1^State Key Laboratory of Pharmaceutical Biotechnology, Li Ka Shing Faculty of Medicine, the University of Hong Kong, Hong Kong SAR, China;

^2^Department of Pharmacology and Pharmacy, Li Ka Shing Faculty of Medicine, the University of Hong Kong, Hong Kong SAR, China;

^3^Department of Medicine, Li Ka Shing Faculty of Medicine, the University of Hong Kong, Hong Kong SAR, China;

^4^Department of Obstetrics and Gynecology, Li Ka Shing Faculty of Medicine, the University of Hong Kong, Hong Kong SAR, China

^*^To whom correspondence may be addressed: Email: [jenseny@hku.hk](mailto:jenseny@hku.hk) and [cwhwoo@hku.
hk](mailto:cwhwoo@hku.hk)

**This file includes:**

Supplementary Figure 1 to 8

Supplementary Table 1

**Other supplementary materials for this manuscript include the following:**

Supplementary Dataset 1 to 2


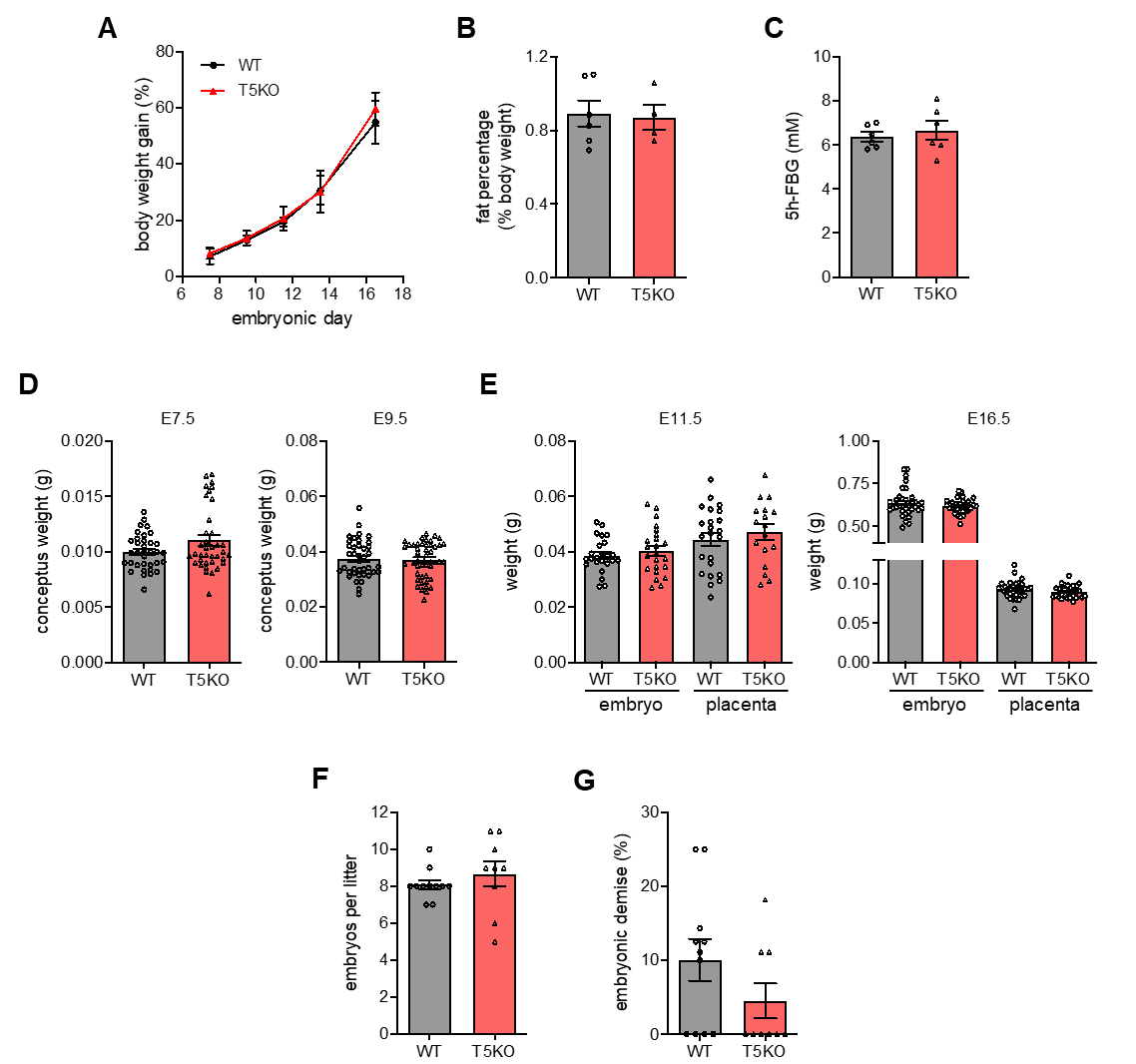


**Supplementary Figure 1 |** TLR5 deficiency did not affect the survival of embryos. Mated wild type (WT) C57BL/6J and TLR5-knockout (T5KO) female mice were sacrificed at indicated gestational days. Presence of vaginal plug was defined as embryonic day 0.5 (E0.5). (**A**) The changes in body weight over the course of pregnancy were recorded (*n*=5-6 mice). (**B**) The weight of visceral fat pad and (**C**) 5-hour fasting blood glucose were measured at E16.5 (*n*=4-6 mice). (**D**) Conceptus weights were measured at E7.5 and E9.5 (*n*=35-37 conceptuses from 4 litters for E7.5; *n*=39-46 conceptuses from 5 litters for E9.5). (**E**) Embryos and placentas were separated at E11.5 and E16.5, and their weights were measured (*n*=17-24 embryos from 4 litters for E11.5; *n*=33-35 embryos from 4 litters for E16.5). (**F**) The litter size and (**G**) the percentage of embryonic demise at E13.5 were determined (*n*=9-11 litters). Data are represented as mean ± s.e.m. The differences between genotypes were determined by two-tailed Student’s *t* test.

**
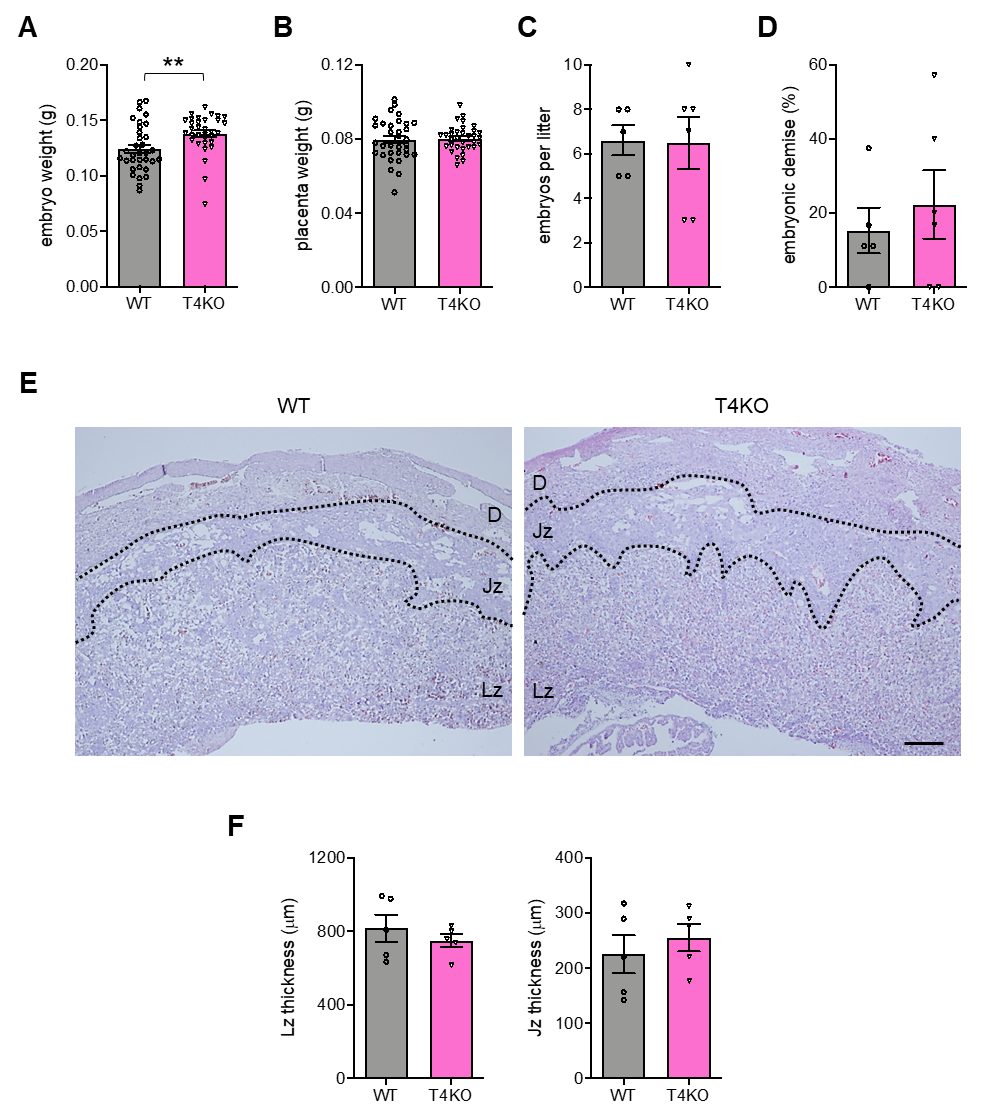
**

**Supplementary Figure 2 |** TLR4 deficiency did not impair the expansion of placental labyrinthine zone. Pregnant wild type and TLR4-knockout (T4KO) mice were sacrificed at embryonic day 13.5. (**A**) Embryo and (**B**) placenta weights were measured (*n*=33 embryos from 4-5 litters). (**C**) Litter size and (**D**) the percentage of embryonic demise were calculated (*n*=5-6 litters). (**E**) Placenta sections were subjected to hematoxylin-eosin staining and representative images are shown. Scale bar is 200μm. Maternal decidua (D), junctional zone (Jz) and labyrinthine zone (Lz) were denoted. (**F**) The thicknesses of Lz and Jz were measured. Each dot represents the average thickness for one litter (*n*=5 litters). Data are represented as mean ± s.e.m. The differences between genotypes were determined by two-tailed Student’s *t* test. ***P*<0.01.

**
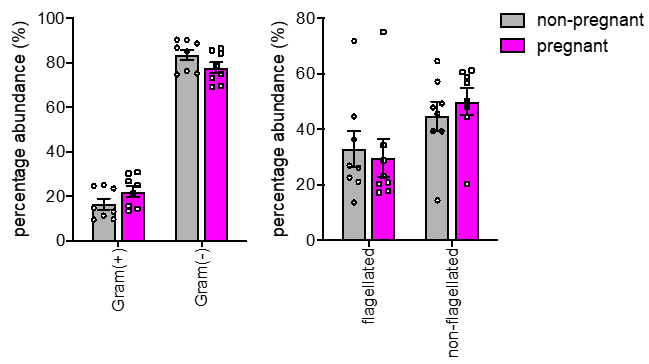
**

**Supplementary Figure 3 |** Pregnancy did not affect the type of gut bacteria. Feces were collected from wild type non-pregnant (non-preg) and pregnant (preg) mice at day 9.5 of gestation and the isolated DNA was subjected to shotgun metagenomic sequencing (*n*=8 mice). The proportions as Gram(+) and Gram(-) bacteria (*left panel*) and the proportions as flagellated and non-flagellated bacteria (*right panel*) were shown. Data are represented as mean ± s.e.m. The differences between non-pregnant and pregnant groups were determined by two-tailed Student’s *t* test.


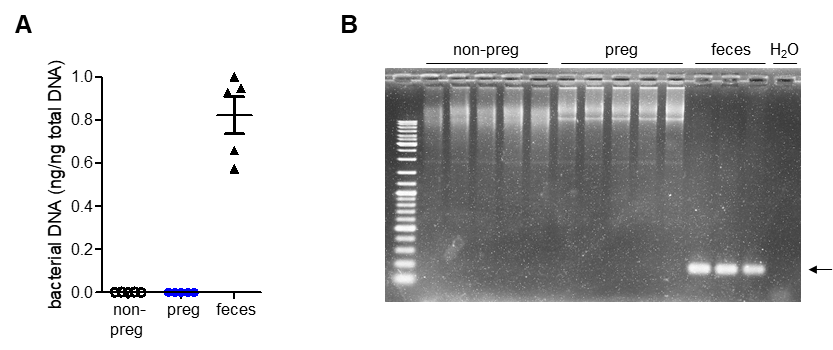


**Supplementary Figure 4 |** Placental microbiome was not observed. Total DNA was isolated from uterus of non-pregnant (non-preg) mice and conceptus from pregnant (preg) mice at embryonic day 9.5 (*n*=5-6 mice). The amplified products of bacterial 16S rRNA gene were separated using agarose gel electrophoresis. Feces was included as positive control. The arrow indicates the target bands.

**
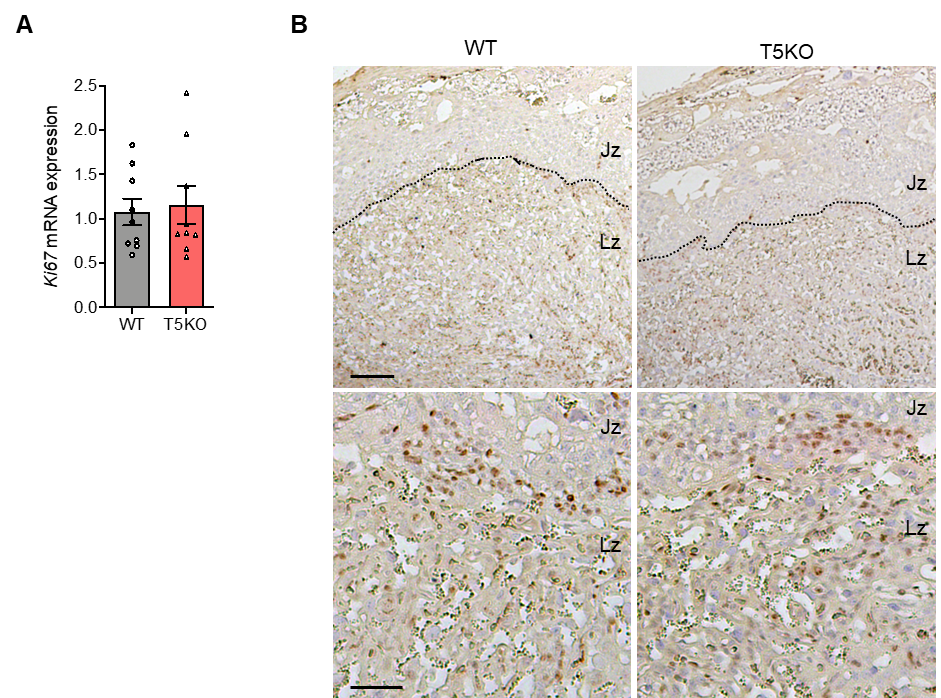
**

**Supplementary Figure 5 |** There was no difference in placental Ki67 expression between wild type and *Tlr5^-/-^* mice. Pregnant wild type and TLR5-knockout (T5KO) mice were sacrificed at embryonic day 13.5 (*n*=9 litters, one randomly picked placenta from each litter). (**A**) The *Ki67* mRNA expression was examined in placenta. (**B**) Placenta sections were subjected to immunohistochemical staining of Ki67 protein. Representative images are shown. Scale bar is 750μm for upper panel and 300μm for lower panel. Junctional zone (Jz) and labyrinthine zone (Lz) were denoted. Data are represented as mean ± s.e.m. The difference between non-pregnant and pregnant groups was determined by two-tailed Student’s *t* test.


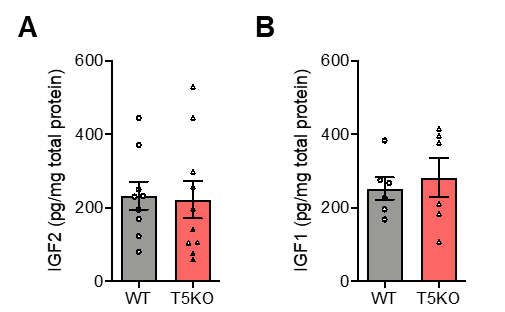


**Supplementary Figure 6 |** TLR5 deficiency did not affect the level of insulin-like growth factors. Conceptuses were isolated from pregnant wild type (WT) and TLR5-knockout (T5KO) mice at embryonic day 9.5. Abundances of (**A**) insulin-like growth factor-2 (IGF2) and (**B**) IGF1 were determined using enzyme-linked immunosorbent assay followed by normalization to the amount of protein loaded (*n*=9-10 litters for *A*; *n*=6 litters for *B*, one randomly picked conceptus from each litter). Data are represented as mean ± s.e.m. The differences between genotypes were determined by two-tailed Student’s *t* test.

**
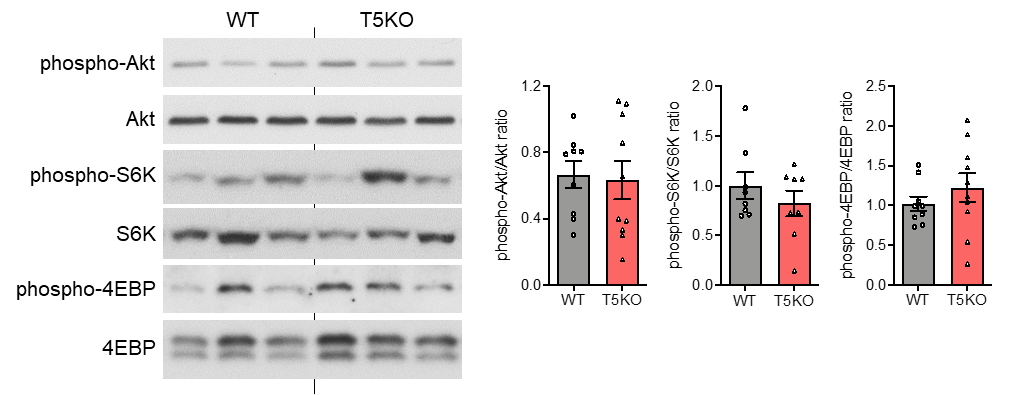
**

**Supplementary Figure 7 |** TLR5 deficiency did not affect phosphorylation of mTOR substrates. Conceptuses were isolated from pregnant wild type (WT) and TLR5-knockout (T5KO) mice at embryonic day 9.5. Protein abundance of phosphorylated and total Akt, S6K, and 4EBP were determined (*n*=9-10 litters, one randomly picked conceptus from each litter). Representative images and the densitometric analyses for immunoblotting are shown. Data are represented as mean ± s.e.m. The differences between genotypes were determined by two-tailed Student’s *t* test.

**
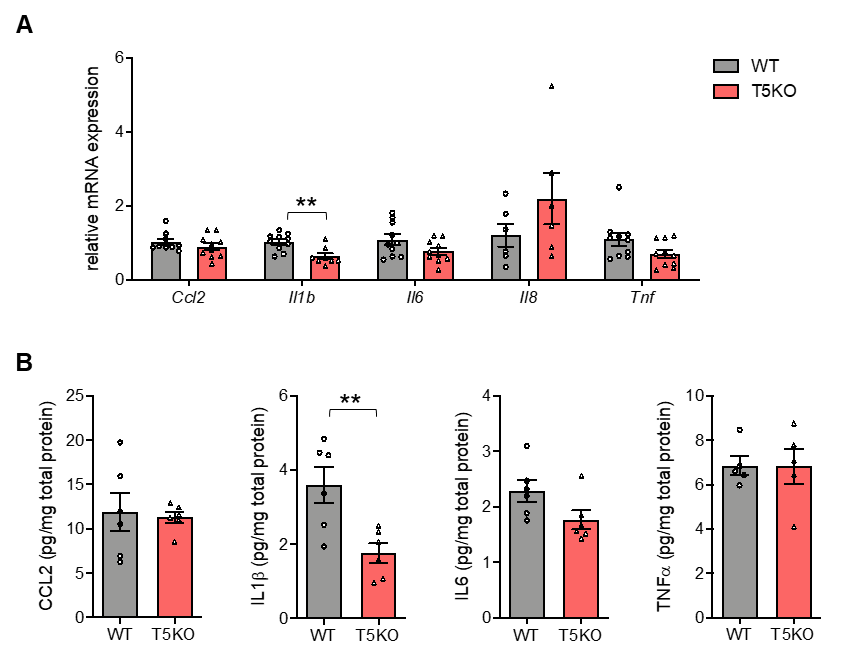
**

**Supplementary Figure 8 |** TLR5 deficiency did not affect proinflammatory cytokine production. Conceptuses were isolated from pregnant wild type (WT) and TLR5-knockout (T5KO) mice at embryonic day 9.5. (**A**) The mRNA expressions of *Ccl2*, *Il1b*, *Il6*, *Il18* and *Tnf* were determined with *Gapdh* as housekeeping control (*n*=8-10 litters, one randomly picked conceptus from each litter). (**B**) The conceptual levels of CCL2, IL1β, IL6 and TNFα were measured using enzyme-linked immunosorbent assays followed by normalization to the amount of protein loaded (*n*=6 litters, one randomly picked conceptus from each litter). Data are represented as mean ± s.e.m. The differences between genotypes were determined by two-tailed Student’s *t* test. ***P*<0.01.

**Supplementary Table 1. Primer sequences for real-time PCR and droplet digital PCR.**

| **Gene** | **Primer sequence** |
| --- | --- |
| *Akt1* (mouse) | 5’-GGCAGGATGTGTATGAGAAGAA-3’ (forward)  5’-AGCGCATCCGAGAAACAA-3’ (reverse) |
| *Ccl2*  (mouse) | 5’-CCCACTCACCTGCTGCTACT-3’ (forward)  5’-TCTGGACCCATTCCTTCTTG-3’ (reverse) |
| *Eif4ebp1* (mouse) | 5’-CGGGAGGAACCAGGATTATCTA-3’ (forward)  5’-TATGAGGCCTGAATGCTGTG-3’ (reverse) |
| *Gapdh* (mouse) | 5’-CTCATGACCACAGTCCATGC-3’ (forward)  5’-CACATTGGGGGTAGGAACAC-3’ (reverse) |
| *Igf1r* (mouse) | 5’-GATTGAGAAGAACGCCGACC-3’ (forward)  5’-GCTTCTCCTCCAATGTCCCT-3’ (reverse) |
| *Il6* (mouse) | 5’-GCCAGAGTCCTTCAGAGAGATA-3’ (forward)  5’-TTTCTGACCACAGTGAGGAATG-3’ (reverse) |
| *Il8* (mouse) | 5’-CCCTGTGACACTCAAGAGCT-3’ (forward)  5’-CAGTAGCCTTCACCCATGGA-3’ (reverse) |
| *Mtor* (mouse) | 5’-CCAAGTGGAGCTGCTTATCA-3’ (forward)  5’-CTGTCCTGGGAACTGAATCAA-3’ (reverse) |
| *Rn18s* (mouse) | 5’-CCCAACTTCTTAGAGGGACAAG-3’ (forward)  5’-CACTAAACCATCCAATCGGTAGTA-3’ (reverse) |
| *Rps6* (mouse) | 5’-TGTTACAGGCCAAGGAGAAC-3’ (forward)  5’-CAGGACACGAGGAGTAACAAG-3’ (reverse) |
| *Rps6kb1* (mouse) | 5’-GTGCCAACCAGGTCTTTCT-3’ (forward)  5’-AGTTGGGTTGTCGGATTGG-3’ (reverse) |
| *Tlr1* (mouse) | 5’-GTCAAGAACATAGGCTGGGTAG-3’ (forward)  5’-GCATGGAACTGGAGGTTTCT-3’ (reverse) |
| *Tlr2* (mouse) | 5’-GAATTGCATCACCGGTCAGA-3’ (forward)  5’-GCCACCAAGATCCAGAAGAG-3’ (reverse) |
| *Tlr3* (mouse) | 5’-TTGTCTTCTGCACGAACCTG-3’ (forward)  5’-CGCAACGCAAGGATTTTATT-3’ (reverse) |
| *Tlr4* (mouse) | 5’-ACATAGATCTGAGCTTCAACCC-3’ (forward)  5’-TGCCATGCCTTGTCTTCA-3’ (reverse) |
| *Tlr5* (mouse) | 5’-AAGTTCCGGGGAATCTGTTT-3’ (forward)  5’-GCATAGCCTGAGCCTGTTTC-3’ (reverse) |
| *Tlr6* (mouse) | 5’-GGCCAACCTTAGAGCTTCAT-3’ (forward)  5’-AGTGACCAACTTCCTCCAATC-3’ (reverse) |
| *Tlr7* (mouse) | 5’-GTTGCGGTACTTAGACTTCTCC-3’ (forward)  5’-GTATCTGTTATCACCGGCTCTC-3’ (reverse) |
| *Tlr8* (mouse) | 5’-GTCTTGACCGTTTGTGGAATG-3’ (forward)  5’-CAGAGGGTAGGTGAGAGAAATG-3’ (reverse) |
| *Tlr9* (mouse) | 5’-ACCTCAGCCACAACATTCTC-3’ (forward)  5’-CGCAGAGATGGTGCAGTATAG-3’ (reverse) |
| *Tnf*  (mouse) | 5’-ACGGCATGGATCTCAAAGAC-3’ (forward)  5’-AGATAGCAAATCGGCTGACG-3’ (reverse) |
| *IL1B* (human) | 5’-GGACAGGATATGGAGCAACAA-3’ (forward)  5’-ACTGGGCAGACTCAAATTCC-3’ (reverse) |
| *GAPDH* (human) | 5’-TGACATCAAGAAGGTGGTGAAG-3' (forward)  5’-CTCTCTTCCTCTTGTGCTCTTG-3' (reverse) |
| *MTOR* (human) | 5’-GCTCATCAAACAAGCGACATC-3' (forward)  5’-ACTGTCCTGGGAACCAAATC-3' (reverse) |
| 16S rRNA (bacteria) | 5’-ACTCCTACGGGAGGCAGCAG-3’ (forward, a.k.a. Eub338)  5’-ATTACCGCGGCTGCTGG-3’ (reverse, a.k.a. Eub518) |
